# Ecological speciation in sympatric palms: 2. Pre‐ and post‐zygotic isolation

**DOI:** 10.1111/jeb.12933

**Published:** 2016-07-20

**Authors:** H. Hipperson, L. T. Dunning, W. J. Baker, R. K. Butlin, I. Hutton, A. S. T. Papadopulos, C. M. Smadja, T. C. Wilson, C. Devaux, V. Savolainen

**Affiliations:** ^1^ Department of Life SciencesImperial College LondonSilwood Park CampusAscotUK; ^2^ Royal Botanic Gardens, KewRichmondUK; ^3^ Department of Animal and Plant SciencesUniversity of SheffieldSheffieldUK; ^4^ Sven Lovén Centre for Marine Sciences, TjärnöUniversity of GothenburgStromstädSweden; ^5^ Lord Howe Island MuseumLord Howe IslandNSWAustralia; ^6^ Royal Botanic Gardens and Domain TrustSydneyNSWAustralia; ^7^ Present address: Department of Animal and Plant SciencesUniversity of SheffieldSheffieldS10 2TNUK; ^8^ Present address: Institut des Sciences de l'Evolution(UMR 5554) CNRS‐IRD‐EPHE‐CIRAD‐University of MontpellierMontpellier34095France

**Keywords:** flowering, hybridization, phenology, post‐zygotic, pre‐zygotic

## Abstract

We evaluated reproductive isolation in two species of palms (*Howea*) that have evolved sympatrically on Lord Howe Island (LHI, Australia). We estimated the strength of some pre‐ and post‐zygotic mechanisms in maintaining current species boundaries. We found that flowering time displacement between species is consistent across *in* and *ex situ* common gardens and is thus partly genetically determined. On LHI, pre‐zygotic isolation due solely to flowering displacement was 97% for *Howea belmoreana* and 80% for *H. forsteriana*; this asymmetry results from *H. forsteriana* flowering earlier than *H. belmoreana* and being protandrous. As expected, only a few hybrids (here confirmed by genotyping) at both juvenile and adult stages could be detected in two sites on LHI, in which the two species grow intermingled (the Far Flats) or adjacently (Transit Hill). Yet, the distribution of hybrids was different between sites. At Transit Hill, we found no hybrid adult trees, but 13.5% of younger palms examined there were of late hybrid classes. In contrast, we found four hybrid adult trees, mostly of late hybrid classes, and only one juvenile F1 hybrid in the Far Flats. This pattern indicates that selection acts against hybrids between the juvenile and adult stages. An *in situ* reciprocal seed transplant between volcanic and calcareous soils also shows that early fitness components (up to 36 months) were affected by species and soil. These results are indicative of divergent selection in reproductive isolation, although it does not solely explain the current distribution of the two species on LHI.

## Introduction

Vicariance can be a major driver of speciation, as geographic barriers isolate populations and reduce gene flow. Nevertheless, divergence of incipient species can occur despite continued gene flow (Dieckmann & Doebeli, 1999; Coyne & Orr, 2004; Smadja & Butlin, 2011), and even without selection (Hoekstra & Coyne, 2007; Devaux & Lande, 2008). Over the last few decades, ecology was shown to play a strong role in initiating speciation (Schluter, 2000, 2001, 2009; Rundle & Nosil, 2005; Nosil, 2012). In particular, several studies have documented speciation with high gene flow (Barluenga *et al*., 2006; Martin *et al*., 2013), with the *Howea* palms of Lord Howe Island (LHI) being one strong convincing example (Savolainen *et al*., 2006). The two species, *Howea forsteriana* and *H. belmoreana*, are endemic to the minute LHI (< 16 km^2^) in the Tasman Sea. They have strongly displaced flowering phenologies and contrasting edaphic distributions. Given these features, ecological speciation was hypothesized to have occurred soon after the deposition of a novel soil type (calcarenite) on LHI (Savolainen *et al*., 2006). However, little is known about the traits involved in pre‐ and post‐zygotic isolation barriers to gene flow, or the intensity and direction of selection acting in this system. Identification of these traits has been successful for many species (Smadja & Butlin, 2011) and is crucial for a better understanding of how speciation proceeded in *Howea*.

In plants, experimental evidence has shown that species boundaries between closely related taxa are largely maintained through pre‐zygotic reproductive barriers (e.g. reviewed in Rieseberg & Willis, 2007; Widmer *et al*., 2009). These barriers arise early during the speciation process and are thought to be more effective in reducing gene flow than post‐zygotic isolating mechanisms (Martin & Willis, 2007; Rieseberg & Willis, 2007; Lowry *et al*., 2008). For sympatric species, pre‐zygotic barriers include gamete and pollen/stigma incompatibilities, pollinator specialization and displacement of flowering times (Gavrilets & Vose, 2007; Rieseberg & Willis, 2007; Lowry *et al*., 2008). From previous studies, we know that *Howea* are wind‐pollinated and that pre‐zygotic reproductive isolation is incomplete (Babik *et al*., 2009), although flowering of *H. forsteriana* occurs several weeks before that of *H. belmoreana* (Savolainen *et al*., 2006). The displacement of *Howea* flowering phenology was documented across LHI, but little is known as to whether the observed displacement is under genetic or environmental control. Flowering time can indeed be plastic along climatic (Franks *et al*., 2007), edaphic (Antonovics, 2006) and density (Mazer & Schick, 1991) gradients. Theory shows that plasticity of flowering time can accelerate and favours speciation under a classical scenario of ecological speciation relying on divergent selection (Gavrilets & Vose, 2007) and that it can be the sole cause of reproductive isolation between populations by substantially reducing pollen flow (Stam, 1983). In addition, soil differences could be linked to post‐zygotic isolation. Indeed, current *Howea* distribution is associated with variation in soil type, pH, water availability and salinity (Savolainen *et al*., 2006; Papadopulos *et al*., 2013, 2014). *Howea belmoreana* is restricted to the volcanic rocks, whereas *H. forsteriana* grows on both calcareous (i.e. calcarenite) and volcanic soils, especially in the south of the island (Papadopulos *et al*., 2013; C. Devaux & V. Savolainen unpublished manuscript). Adaptation of species to different soil types has not yet been evaluated, nor has the distribution, frequency and fitness of hybrids.

To evaluate the role of pre‐ and post‐zygotic barriers in the current distribution of *Howea*, and validate previous assumptions, we estimated some key elements of the *Howea* ecological speciation scenario. We evaluated whether genetic vs. plastic effects contribute to the displacement of *Howea* flowering phenologies and quantified the strength of pre‐zygotic reproductive isolation *in situ* and *ex situ* in common gardens. We also measured the strength of post‐zygotic barriers to gene flow by (i) examining the occurrence of hybrids at early and late stages in two sites in which we expect hybridization to be higher than elsewhere on the island and (ii) estimating early fitness components of the two species in reciprocal seed transplant experiments between the two main soil types (volcanic vs. calcarenite) on LHI and under controlled conditions in a glasshouse in the UK.

## Materials and methods

### Estimates of pre‐zygotic reproductive isolation

**Figure 1:**
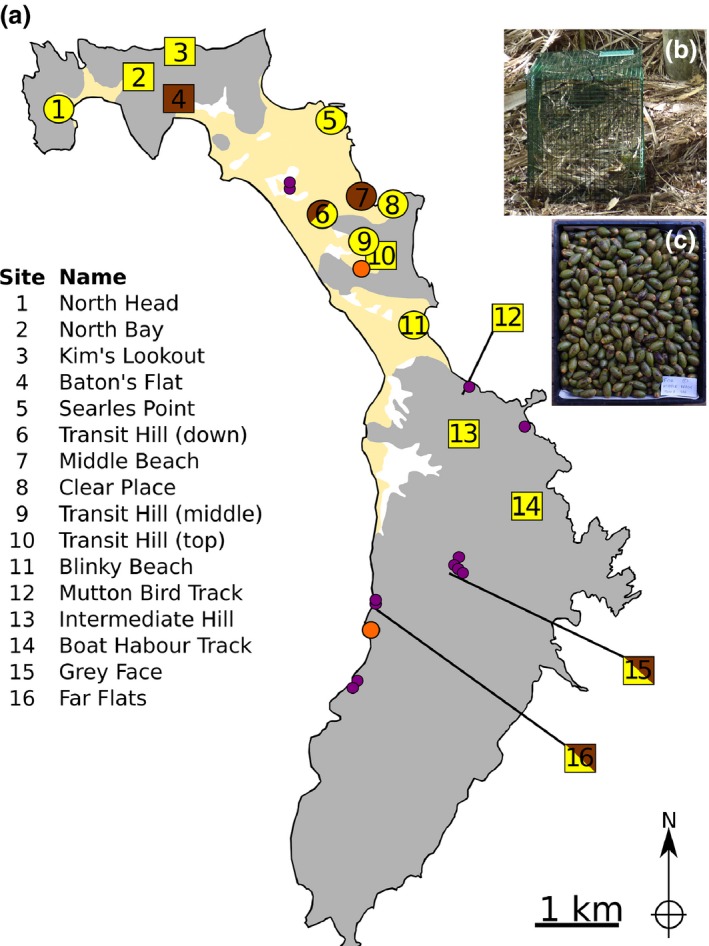
Reciprocal seed transplant experiment on Lord Howe Island (panel a). Five sites that provided seeds are brown, and 14 sites that were the location of the transplant experiments are yellow (circles on calcareous soil, squares on volcanic soil). Yellow background indicates calcareous soil, and grey indicates volcanic soils and white indicates alluvial soils. An example seed cage (panel b) and seed collection (panel c) is shown. The location of adult hybrids (purple circles) and juvenile palms (orange circles) sampled for AFLP genotyping is also shown. Adult hybrids were only located where both *Howea belmoreana* and *H. forsteriana* grow together, and always on volcanic or alluvial soils, and not on calcareous soils. The juvenile sampling location of Far Flats in the South was on volcanic soil, and Transit Hill in the North spanned volcanic and calcareous soils.

To test for genetic and plastic effects on *Howea* flowering phenology, and most importantly their displacement, we monitored the flowering phenology of *H. belmoreana* and *H. forsteriana* on a weekly basis at two different sites: (i) at a single volcanic site on LHI (the Far Flats; Lat. −31.565, Long. 159.076 decimal degrees, site 16 in Fig. 1) on 100 trees (42 *H. belmoreana*, 54 *H. forsteriana* and 4 F1 hybrids, see below for the genetic confirmation of their status) from August to December 2009 and (ii) at the Royal Botanic Gardens and Domain Trust, Sydney (Lat. −33.866, Long. 151.217 decimal degrees) on 99 palms (27 *H. belmoreana* and 72 *H. forsteriana*) from August to November 2011. Unusually for palms, *Howea* are structurally protandrous at the level of the inflorescence: each inflorescence produces unisexual flowers that are male in a given year and female the year after, and trees bear dozens of inflorescences of different ages (and thus sexes). For each flowering period and each site, phenology was monitored at the inflorescence level, counting inflorescences with open male or female flowers for each tree. These data provide estimates of dichogamy (the displacement of male and female flowering times) for each location, and displacement of flowering phenology at the species (here population) level, and thus the intensity of pre‐zygotic reproductive isolation between the two species. With these estimates, we can quantify the effect of dichogamy (most probably protandry of *H. forsteriana*, the maturation of male flowers before female flowers within a given year, as in Savolainen *et al*., 2006) on reproductive isolation and address the plastic component of the flowering displacement between species.

Dichogamy *D* within species was estimated by modifying equation 3 of Devaux & Lande (2009); it assumes equal production of pollen among inflorescences and accounts only for temporal differences in the production of male and female inflorescences over the entire flowering period of each species:

$$D=0.5-\frac{\sum_{w}I_{fw}H_{fw}}{\sum_{w}I_{fw}},H_{fw}=\frac{I_{mw}}{I_{fw}+I_{mw}}$$

with *H*_*fw*_ the proportion of pollen received in week *w* of the flowering period, and *I*_*fw*_ and *I*_*mw*_ the number of inflorescences bearing female and male flowers in week *w*, respectively. *D *=* *0.5 when male and female flowering times are completely displaced (maximal value; complete asynchrony), and *D *=* *0 when male and female flowering time fully overlap (minimal value; complete synchrony). Note that *D* does not discriminate between the type of dichogamy (protandry vs. protogyny), but the direction of dichogamy is unequivocal in our data as at both sites male flowering begins before female flowering at the population/species level.

The strength of a pre‐zygotic isolating barrier between focal species *i* and the other species *j* was estimated on LHI and in Sydney with *RI*_*i*_, which integrates the displacement in flowering phenology between species *i* and *j* over weeks (*w*) of the flowering season, following equation 3 by Devaux & Lande (2009):

$$RI_{i}=1-\frac{\sum_{w}I_{ifw}H_{iw}}{\sum_{w}I_{ifw}},H_{iw}=\frac{I_{jmw}}{I_{imw}+I_{jmw}}$$

with *H*_*iw*_ the proportion of heterospecific pollen received by species *i* during week *w*, and *I*_*ifw*_ and *I*_*imw*_ the number of open inflorescences of sex *f* (female) or *m* (male) of species *i*, respectively, during week *w*.

The *RI* index was calculated for each species as gene flow is expected to be asymmetrical due to differences in dichogamy between species at the population level. The 95% confidence intervals (CI) for both *D* and *RI* estimates were calculated by bootstrapping over *Howea* individuals using 5000 iterations in R (version 3.2.2, https://www.rproject.org).

For comparative purposes, some of these indices were also applied to the phenology data in Savolainen *et al*. (2006).

### Estimates of post‐zygotic reproductive isolation

#### Hybrid palm prevalence at juvenile and adult life stages

To establish whether and when post‐zygotic selection acts, we evaluated the occurrence of hybrid individuals at early and late life stages in two sites in which we expect hybridization to be important: the Far Flats (−31.568, 159.076; site 16 on Fig. 1), in which the two species grow intermingled on volcanic soil, and Transit Hill (−31.534, 159.074; sites 9 & 10 on Fig. 1), in which the two species grow adjacently, with *H. belmoreana* on volcanic soil and *H. forsteriana* on calcareous soil. Hybrid identification in the field can only be attempted at the adult stage using, as a main criterion, intermediate leaf morphology between the two parental species. To detect hybrids accurately, we therefore used Amplified Fragment Length Polymorphism (AFLP) genotypes of dried leaf samples collected in the two sites above from (i) randomly sampled seedling individuals, (ii) four morphologically identified hybrid adult trees in the Far Flats (we could not find morphological hybrids in Transit Hill) and (iii) 131 previously randomly sampled adult trees (Savolainen *et al*., 2006; Babik *et al*., 2009; Papadopulos *et al*., 2013). We also included genotypes and re‐assigned adult trees previously sampled across LHI in 31 locations (404 trees, previously assigned to 215 *H. forsteriana*, 184 *H. belmoreana* and five hybrids; Papadopulos *et al*., 2013). Leaves of 142 seedlings were randomly collected at the Far Flats in June 2010 in a 50 × 50 m quadrat, and leaves of 81 seedlings were randomly collected at Transit Hill in October 2010 in a 30 × 30 m quadrat spanning both volcanic (56 seedlings) and calcareous (25 seedlings) soils (Table S1, Fig. 1). The leaves of 85 and 46 adult trees were randomly sampled in the Far Flats and Transit Hill, respectively, from 2008 to 2010. Those trees grow about 200–300 m from the sampling quadrats in the two sites (Fig. 1). Including the same individuals as in the previous studies enabled us to validate the genotyping and assigning methods, get an estimate of the distribution of hybrid ages (from first to fourth generation hybrid classes) across LHI and have a better representation of adult genotypes of pure species for the assigning method. To complement our database and have better estimates of the occurrence of hybrids on the island, we extensively searched across the island for new morphological hybrid trees during three field trips spanning from 2009 to 2010. We found 12 new morphological intermediate adult trees, which we genotyped using AFLPs (locations on Fig. 1).

DNA was extracted from silica dried leaf samples, and AFLP genotypes were generated using three primer combinations (B12, G7 and G21) as described in Papadopulos *et al*. (2013). The same AFLP bin sets (i.e. fragment length classes) used in Papadopulos *et al*. (2013) and positive control samples were included to ensure consistency between these new data and the previously generated 404 AFLP profiles that we also use here. We assigned the genetic background of each AFLP‐genotyped individual as either pure species or hybrid among four classes (F1, F2 or backcross to either species) using a Bayesian clustering approach of multiple and unlinked markers implemented in *NewHybrids* (version OSX). This method uses the AFLP genotypes to calculate for each sample the proportion of each parental species, and the percentage of heterozygous loci. From these data, a posterior probability is generated for each of the six distinct genotype classes (Anderson & Thompson, 2002). To allow detection of fourth generation hybrids and reduce the risk of false assignment to F1, we used the 45‐class approach as described in Milne & Abbott (2008). Individuals were unambiguously assigned to one of the six genetic backgrounds if they had a posterior probability above 95% for that class. If there was no clear optimum, the cumulative probability was used to assign the individual to an indeterminate hybrid class, or as uncertain if pure and hybrid classes were equally likely.

#### Soil type adaptation of pure species and hybrid seedlings

We evaluated local adaptation to soil type and post‐zygotic isolation at an early stage in *Howea* by conducting reciprocal seed transplant experiments between two soil types on LHI, as well as under controlled glasshouse conditions at Imperial College London (UK). We distinguished on purpose *H. forsteriana* growing on calcarenite (*FC*), and *H. forsteriana* growing on volcanic soil (*FV*) in the experimental design to assess their potential adaptation to calcareous and volcanic soils, respectively. Seeds for both experiments were collected on LHI by local palm seed harvesters (R. Byrne and L. Wilson) in June 2010. To ensure a representative sample of genotypes, seeds were sampled from multiple individuals and locations. For *H. belmoreana* (*B*), *H. forsteriana* growing on calcarenite (*FC*) and *H. forsteriana* growing on volcanic soil (*FV*), seeds were collected from at least two trees from five locations (Fig. 1a). Seeds were also collected from three morphological F1 hybrid trees from a single site (*H*, from site 15, Fig. 1a). All hybrids and a seedling from each collected tree were AFLP‐genotyped, as described above, to confirm their genetic identification.

Seeds were fumigated using 330 g/kg phosphine and planted at 14 transplant sites across the island, including six on calcareous soil (Fig. 1a, circles) and eight on volcanic soil (Fig. 1a, squares). To protect them from rat predation, seeds were planted in wire mesh cages (30 × 30 × 50 cm, length × width × height, Fig. 1b) that were buried approximately 10 cm into the ground. At each of the 14 sites, 10 cages were set up: three containing only *B* seeds, three containing *FC* seeds, three containing *FV* seeds and one containing *H* seeds. Each cage contained 24 seeds consisting of a random sample of the progeny from at least two trees from two locations, except for *H* seeds that all came from the same location (3360 seeds in total). Germination, survival and height of all individuals were monitored monthly from planting in June 2010 until June 2013.

A reciprocal seed transplant experiment was repeated in the UK. The LHI soil types were mimicked using a combination of commercially available soils. For calcarenite, we mixed 80% of coral sand (Unipac, Northampton, UK), 20% of seed sowing compost (John Innes, Norwich, UK) and 40 mL of dolomitic lime (Francis Flower, Wicken, UK) per litre of soil mix. For volcanic, we mixed 60% of ericaceous compost (J. Arthur Bower, Woolsack Way, UK), 20% of Irish moss peat (Westland, Thatcham, UK) and 20% perlite (Sinclair, Woolsack Way, UK). In September 2010, we planted 2088 seeds in 90 trays across three spatial blocks, using one seed per 100 mL cell in each tray of 24 seeds. Soil type was randomly assigned to each of the 90 trays (45 for each) and seeds from collected trees were randomly assigned among trays, ensuring a single seed source per tray (*B*,* FC*,* FV* or *H*) and according to the number of seeds available per tree. In total, 288 *H* seeds and 600 seeds each of *B*,* FC* and *FV* seeds were planted (Table S2). Prior to planting, all seeds were weighed (precision ± 0.1 g) and their length and width measured (precision ± 1 mm). Cells in each seed tray were two‐thirds filled with soil and the seed sown ~ 18 mm from the top. Due to compaction over time, both soil types were topped up in March 2011. We recorded pH, with both kits (Gardman, Peterborough, UK) and colour strips (Sigma, London, UK), in three random calcareous and three random volcanic half trays (72 cells in total) left empty on purpose, at days 0, 48, 153 and 192 after sowing. Artificial ‘volcanic soil’ had a mean pH of 6 (SD = 0), and ‘calcareous soil’ had a mean pH of 7.81 (SD = 0.26), consistent with measures from LHI (Savolainen *et al*., 2006). The glasshouse temperature was set to remain between 17 and 22 °C, to reflect LHI mean minimum and maximum annual temperatures over the past 24 years. Supplementary lighting was automatically switched on from 6 am to 6 pm if the natural light fell below a certain threshold. Watering of the trays occurred every 10 days. The trays were also placed upon ‘Florimat’ capillary matting to maximize water retention. Germination of seeds as well as survival and height of seedlings were monitored every 10 days from planting in September 2010 until September 2013.

We built different statistical models to evaluate local adaptation to soil type of the three pure species classes and to post‐zygotic isolation. Local adaptation was assessed analysing variations in the binary responses of germination and survival at month 36 on LHI (i.e. germinated/not germinated; survived/not survived). We used generalized linear mixed models including as explanatory variables the seed source (with the three pure species levels *B*,* FV*,* FC*), the soil type on which seeds were planted (volcanic V vs. calcareous C) and their interaction as fixed effects. We also accounted for transplant location nested into soil type, and cage replicates nested within transplant location as random factors. For both analyses, we assumed binomial distributions for the number of germinated seeds and alive seedlings (GLIMIX function; SAS 9; Cary, NC, USA). We built the same models separately for the *H* seeds, removing the random cage factor. Because sample sizes were unbalanced (e.g. across soil types), all effects were tested using Type III errors. If the interaction between seed source and soil type was significant in the models, we further performed pairwise comparisons on all seed source by soil type combinations (LSMEANS function combined with a Tukey adjustment; SAS 9). All generalized models were validated as their dispersal estimates were below 1. Logistic curves were fitted to cumulative germination over time for each of the six treatments crossing the three pure species classes and the two soil types (NonlinearModelFit, Mathematica 8; Hanborough, UK), and likelihood ratios were used to test for their effects and select the most appropriate model (Fig. S1 and Tables S4 and S5). We did not try fitting curves at the location or cage level to avoid losing statistical power. We could not accurately analyse variation in survival for germinated seeds only (i.e. independently of germination) and variation in height of the surviving seedlings, as the sample sizes for each treatment were very unbalanced (from 3 to 45); therefore, we built only Type I error generalized models including the above three fixed factors, and giving the soil effect the highest potential for significance.

We used an under‐sampling method to assess post‐zygotic isolation because hybrid seeds were three times fewer than seeds from each pure species class. We therefore balanced the experimental protocol by randomly sampling without replacement 24 replicate (equivalent to one cage) data points for each of the three pure species classes and each location. We also balanced replication level for the effect of soil type by sampling six locations among the eight available for the three pure species classes and the hybrid class. In total, we had 24 seeds × 4 classes × 2 soil types × 6 locations = 1152 data points (from the original 3360). For each of 1000 samples, variations in germination and survival were analysed as above, except that the location was the only random factor.

We used similar statistical models to analyse the UK data. In models analysing variations in germination and survival, we included the same aforementioned fixed effects of seed source and soil type, but also the residuals of seed weight regressed linearly on its volume (approximated by a cylinder) as covariates to control for seed density. We also accounted for spatial blocks, as they were differentially affected by sun scorching, and position of trays (edge vs. centre) within blocks as fixed effects, and replicate trays as a random factor.

## Results

### Strong pre‐zygotic reproductive isolation

**Figure 2:**
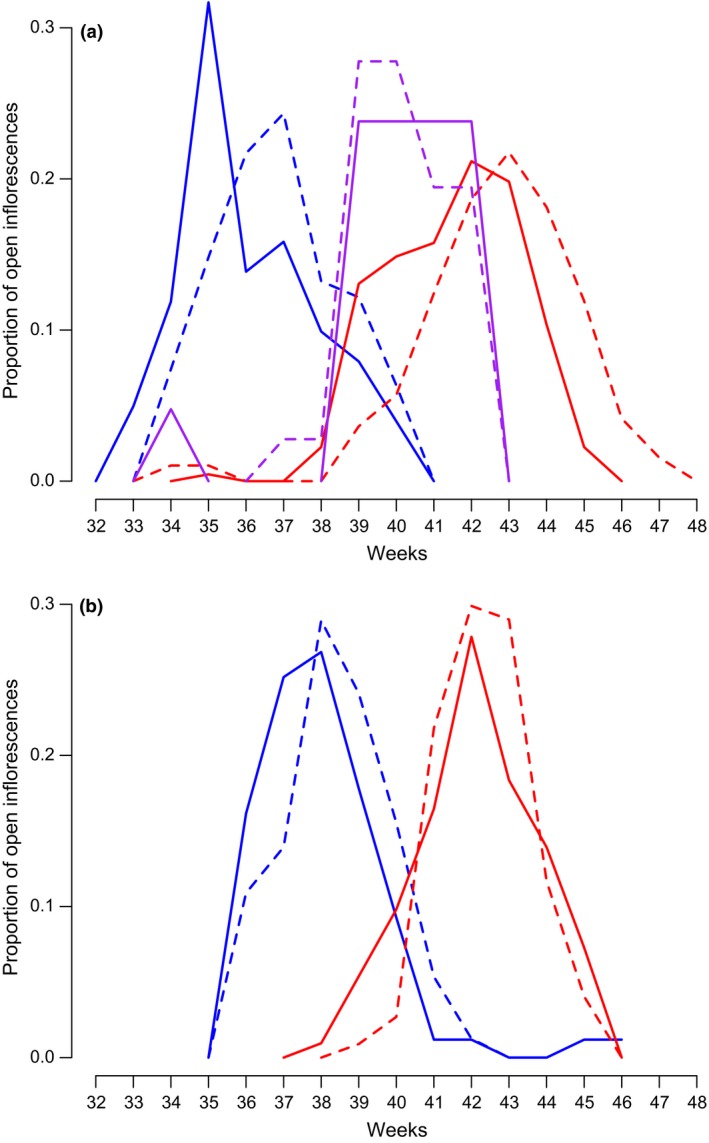
Flowering phenology, shown as weeks of the year, of each *Howea* species is strongly displaced on (panel a) Lord Howe Island (LHI) and in (panel b) Sydney; *Howea belmoreana* in red (LHI male number of flowering trees *n* = 39, female *n* = 30, Sydney male *n* = 24, female *n* = 23); *H. forsteriana* in blue (LHI male *n* = 42, female *n* = 36, Sydney male *n* = 42, female *n* = 57); hybrids palms in purple (LHI male *n* = 2, female *n* = 3); male and female phases in solid and dotted lines, respectively.

When measured at the population level on LHI, dichogamy was higher for *H. forsteriana,* which was protandrous (*D*_*F*_ = 0.18, CI 0.08–0.24), than it was for *H. belmoreana*, which had synchronous male and female flowering (*D*_*B*_ = 0.04, CI 0.04–0.10) In Sydney, we also found both species to be protandrous, although bootstrap support indicates no difference from synchrony (*D*_*F*_ = 0.10, CI 0.00–0.17; *D*_*B*_ = 0.11, CI 0.01–0.18). The flowering phenologies of the two species at both sites were strongly displaced, with peak flowering times shifted by a few weeks (Fig. 2). The displacements of phenologies were consistent across locations (Sydney and LHI), with *H. forsteriana* flowering earlier in the season than *H. belmoreana*. Assuming no other isolating mechanisms, pre‐zygotic isolation through the sole displacement of flowering phenologies was strong, that is between 0.80 and 0.97 depending on location and direction of the cross. During the season monitored, *H. belmoreana* was more reproductively isolated (*RI*_*B*_ = 0.97, CI 0.87 –1.00, on LHI and 0.94, CI 0.77–0.99, in Sydney) from *H. forsteriana* than *H*. *forsteriana* was isolated from *H. belmoreana* (*RI*_*F*_ **= 0.80, CI 0.64–0.89, on LHI and 0.82, CI 0.63–0.92, in Sydney). Although confidence intervals of reproductive isolation estimates are large, these estimates indicate that displacement of flowering phenology can lead to asymmetrical pollen flow between the species: *H. forsteriana* was estimated to have received more heterospecific pollen than did *H. belmoreana*, and this pattern is explained by *H. forsteriana* flowering earlier and being more protandrous compared with *H. belmoreana*; that is, the early *H. forsteriana* male flowers were unlikely to pollinate the late *H. belmoreana* female flowers.

The flowering phenology of three hybrid palms was also monitored (but one did not flower during the study period). They all bore female inflorescences (samples 635, 636 and 637), and two also bore male flowers (samples 635 and 636, Table S1). Although here confirmed as hybrids, their classification as either F1, F2 or backcrosses was not conclusive (Table S1): F1 status reached the highest probability for trees 635 and 636 (55% and 54%, respectively), and F2 status reached the highest probability for tree 637 (66%). The flowering phenology of two hybrids (635 and 637) was intermediate between *H. forsteriana* and *H. belmoreana*. Hybrid tree 636 had a female flowering phenology intermediate between *H. forsteriana* and *H. belmoreana*, whereas the male flowering time coincided with that of *H. forsteriana* (Fig. 2a).

### Rare occurrence of hybrids on LHI

We confirm the accuracy of the species assignment method used here, despite having fewer AFLP markers than in previous studies: 398 of the 404 palms genotyped agreed with previous estimates (Papadopulos *et al*., 2013), whereas the remaining six individuals lacked statistical support and could not be assigned by *NewHybrids* (Table S1). The five palms genetically identified as hybrids in Papadopulos *et al*. (2013) were also assigned as hybrids here (samples 635–639, Table S1). These results show that we can confidently use the assignment method to estimate hybridization at an early stage as done here. Among the 12 new, morphologically intermediate trees found on LHI, 11 were assigned to F1 hybrids, and one was found to be in fact a pure *H. forsteriana* (sample 228, Table S1).

Among the 223 seedlings sampled in two sites in which *H. belmoreana* and *H. forsteriana* grow together, 83% were assigned to pure species, 7% to hybrid classes and 10% remained ambiguous (Table 1). Most of the unassigned individuals grew in the intermingled population (18/142 = 13% in the Far Flats vs. 5/81 = 6% in Transit Hill), whereas most of the detected hybrids grew in the population in which the species grew adjacently (14/81 = 17% in Transit Hill vs. 1/142 = 0.7% in the Far Flats). All but one hybrid were assigned to classes of second or later generations (Table 1). Despite intensive searches of the area, we could not detect morphologically intermediate trees in Transit Hill. In contrast, four such intermediate trees were found at the Far Flats, including one F1 hybrid and three of later generations (Table 1).

**Table 1:** Genetic assignments provided by *NewHybrids*. Adult trees from Transit Hill and Far Flats are included in the adults from across Lord Howe Island (LHI)

	Adults from across LHI	Adults from Transit Hill only	Juveniles from Transit Hill	Adults from Far Flats only	Juveniles from Far Flats
Nonhybrid (pure species)	394	45	62	80	123
F1	13		1	1	
F2					
Backcross			2		1
F1 or F2			1		
Backcross or F2	1		10	1	
F1, F2 or backcross	2			2	
No confident assignment	6	1	5	1	18

### Evidence for adaptation to soil type

**Figure 3:**
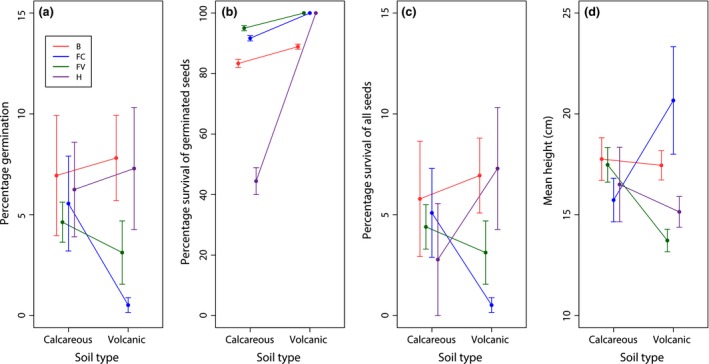
Germination (panel a), survival of germinated seeds (panel b), survival of all seeds (panel c) and height (cm) of seedlings (panel d) across soil types (C = calcarenite, V = volcanic) from the reciprocal transplant experiment on Lord Howe Island (LHI). *Howea belmoreana* (*B*), *H. forsteriana* from calcareous soils (*FC*), *H. forsteriana* from volcanic soils (*FV*) and hybrids (*H*). Each point represents the mean (± SE) percentage germination or survival (panels a–c) or seedling height (panel d) across replicate transplant sites for calcareous (six sites) and volcanic (eight sites) soil. Sample sizes for germinated seeds (panel a): *B *=* *75 (30 C, 45 V), *FC* = 27 (24 C, 3 V), *FV* = 38 (20 C, 18 V), *H *=* *23 (9 C, 14 V); and for survived seeds (panels b, c, d): *B *=* *65 (25 C, 40 V), *FC* = 25 (22 C, 3 V), *FV* = 37 (19 C, 18 V), *H *=* *18 (4 C, 14 V).

Firstly, we confirm the genetic identity of the trees used for seed collection (samples 640–650 were pure species and samples 230–233 were F1 hybrids; Table S1). On LHI, seeds started to germinate 15 months after planting, reaching a total of 140 (4.6%) germinations from seeds collected on pure species trees and 23 (6.8%) from seeds collected on F1 trees (Fig. 3a). By the end of the 36‐month experiment, 91% of the germinated seeds from pure species trees and 78% of the seeds from F1 hybrid trees had survived (Fig. 3b). Most of the seedling deaths occurred during a severe drought in December 2012, with 13 dead seedlings on calcareous soils and five dead seedlings on volcanic soils (Fig. 3b, c), resulting, however, in no difference between soil types in overall survival of seedlings (*P *=* *0.158, Table 2). These overall results hide large differences between species in germination, survival and height of seedlings on volcanic vs. calcareous soils, as shown by the significant effects of seed sources and its interaction with the soil on which seeds were planted for these three early‐stage fitness components (Table 2).

**Table 2:** Results of the generalized mixed models to test the effects of seed origin and soil on germination, seedling survival and seedling height on Lord Howe Island

	Germination^a^				Survival^b^				Height			
Effect	F	Numerator DF	Denominator DF	*P*	F	Numerator DF	Denominator DF	*P*	F	DF	Type III SS	*P*
Soil type	3.51	1	12	0.086	2.26	1	12	0.158	0.07	1	1.4	0.792
Seed source	8.58	2	2898	**< 0.001**	6.21	2	2898	**0.002**	2.75	2	110.3	0.068
Soil type × Seed source	5.24	2	2898	**0.005**	5.07	2	2898	**0.006**	4.25	2	170.4	**0.016**

Wald F test statistic values are reported, along with the degrees of freedom (DF) and associated *P* values. Type III errors were used due to unbalanced sample sizes, and Type III sums of squares (SS) are also reported for the seedling height model. Site and cage replication were modelled as random effects, and the size of these is reported. *P *<* *0.05 are highlighted in bold.

a Random effect estimates (and standard error): transplant location nested into soil type = 0.496 (0.317); cage replicates nested within transplant location = 0.558 (0.207).

b Random effect estimates (and standard error): transplant location nested into soil type = 0.648 (0.402); cage replicates nested within transplant location = 0.596 (0.220).

The numbers of seedlings alive from *H. belmoreana* trees (*B*) and *H. forsteriana* trees collected on volcanic soil (*FV*) were not affected by the soil type on which they were planted (Table 3B). However, when grown on volcanic soil *B* seeds germinated earlier and at a higher rate than did *H. forsteriana* seeds (Fig. S1a), but they did not survive as well as *H. forsteriana* seeds (Fig. 3b). This differential death rate of germinated seeds did not compensate for the difference in germination, and at the end of the experiment, more seedlings of *B* than of *FV* were alive (*P *=* *0.015, Fig. 3c and Table 3B) and they were taller (Fig. 3d), suggesting a better early fitness component of *H. belmoreana* than *H. forsteriana* from volcanic soils (*FV*) when growing on volcanic soils. In contrast, *FC* germination and survival were strongly affected by the soil on which seeds were planted: these early fitness components were much lower on volcanic than on calcareous soil (*P *<* *0.01, Table 3B, Fig. 3a and S1a) and also much lower compared with both *B* (*P *<* *0.001) and *FV* on volcanic soils (*P *<* *0.02, Table 3B). This indicates that the *FC* class is better adapted to calcareous than volcanic soils. At the end of the experiment, *B* and *FC* seedlings were similarly tall and taller than *FV* seedlings on volcanic soils (Fig. 3d), but note that estimates of *FC* seedling height were from a small sample size (*n* = 3). Germination, survival and height of surviving seedlings from *FC*,* B* and *FV* seeds were similar on calcareous soils (Fig. 3, Table 3b).

**Table 3:** Results of the pairwise comparisons to test the effects of seed origin (A) and the interaction between seed origin and soil (B) on seed germination and seedling survival rates on Lord Howe Island

Effect		Germination				Survival			
		Estimate	Standard Error	*t*	*P*	Estimate	Standard Error	*t*	*P*
(A) Seed effects									
*B*	*FC*	1.479	0.381	3.88	**< 0.001**	1.348	0.392	3.44	**0.001**
*B*	*FV*	0.717	0.282	2.55	**0.011**	0.540	0.295	1.83	0.067
*FC*	*FV*	−0.762	0.400	−1.91	0.057	−0.807	0.407	−1.99	**0.047**
(B) Interaction effects									
*B* C	*FC* C	0.254	0.402	0.63	0.527	0.115	0.431	0.27	0.790
*B* C	*FV* C	0.343	0.408	0.84	0.401	0.104	0.433	0.24	0.810
*B* C	*B* V	−0.156	0.527	−0.30	0.767	−0.305	0.589	−0.52	0.605
*B* C	*FC* V	2.547	0.770	3.31	**0.001**	2.275	0.812	2.80	**0.005**
*B* C	*FV* V	0.934	0.569	1.64	0.101	0.671	0.626	1.07	0.283
*FC* C	*FV* C	0.088	0.420	0.21	0.834	−0.011	0.439	−0.02	0.981
*FC* C	*B* V	−0.411	0.537	−0.76	0.445	−0.420	0.593	−0.71	0.479
*FC* C	*FC* V	2.292	0.776	2.95	**0.003**	2.160	0.815	2.65	**0.008**
*FC* C	*FV* V	0.680	0.578	1.18	0.240	0.556	0.630	0.88	0.377
*FV* C	*B* V	−0.499	0.540	−0.92	0.356	−0.409	0.591	−0.69	0.489
*FV* C	*FC* V	2.204	0.778	2.83	**0.005**	2.171	0.814	2.67	**0.008**
*FV* C	*FV* V	0.592	0.581	1.02	0.308	0.567	0.627	0.90	0.366
*B* V	*FC* V	2.703	0.647	4.18	**< 0.001**	2.580	0.654	3.95	**< 0.001**
*B* V	*FV* V	1.091	0.388	2.81	**0.005**	0.976	0.400	2.44	**0.015**
*FC* V	*FV* V	−1.612	0.680	−2.37	**0.018**	−1.604	0.685	−2.34	**0.019**

Pairwise comparisons were performed using the LSMEANS function in SAS, and the estimate of least squares means, standard error, *t*‐test and *P* values are reported. The seed origins tested were *Howea belmoreana* (*B*), *H. forsteriana* from calcareous soils (*FC*) and *H. forsteriana* from volcanic soil (*FV*). The interaction between seed source and soil (part B) is a pairwise comparison of all combinations of each of the three seed sources growing on either volcanic (V) or calcareous (C) soil. *P *<* *0.05 are highlighted in bold.

On LHI, seeds collected on F1 hybrid trees germinated equally well on calcareous and volcanic soils ($X_{1}^{2}=0.03$, *P *=* *0.85), Fig. 3a). Survival of *H* seedlings was strongly affected by the soil on which they grew: 100% of germinated seedlings survived on volcanic soils, whereas only 44% survived on calcareous soil ($X_{1}^{2}=11.72$, *P *=* *0.0006), Fig. 3b, c). These results indicate a better adaptation to volcanic than calcareous soils, as they were provided by only 23 germinated seeds, and once grown, the *H* seedling reached the same size on the two soil types at the end of the 36th month (*P *=* *0.1, Fig. 3d).

Seed sources and soil types on which seeds were planted affected germination and survival differently in the UK glasshouse compared with LHI. In the UK, seeds started to germinate earlier than they did on LHI (6 months after sowing), germination was higher than on LHI (13.7%), but survival was much lower than on LHI (< 50%, and down to 5%, Fig. S2 and Table S3). All effects included in the model significantly affected germination, except the seed source and the interaction between seed source and soil type (Table S3), which were highly significant for the LHI data (Table 2). Seed volume was strongly correlated to seed weight and explained 70% of its variation for *B* seeds, 65% for *FV* seeds and 55% for *FC*.

When comparing germination or survival among the seeds from three pure species classes and the hybrid class in a balanced design, evidence for post‐zygotic isolation remains inconclusive. This absence of effect most probably results from too low sample sizes; detecting that the *H* seeds survived less than did the seeds from any of the parental species with 80% power, and using the estimates found here, would require measuring survival of 600 seeds and 900 seeds for comparison with *B* and *FC* seeds on calcareous soils, respectively, and 67 000 seeds and 350 seeds for comparison with *B* and *FV* seeds on volcanic soils. *P*‐values obtained from the under‐sampling method are nevertheless indicative of a seed source by soil type interaction: 11% of the *P*‐values for the fixed seed source effect were below 5%, 0.3% for the soil effect were below 5%, whereas 40% for the seed source by soil type interaction were below the 5% threshold and half of them were below the 10% threshold (Table 4). These *P*‐values match the raw estimates of survival for the four seed classes, with *H. belmoreana* seeds and seeds from hybrids performing equally on volcanic soils, seeds of *H. belmoreana* and *H. forsteriana* performing equally well on calcareous soils, seeds of *H. forsteriana* from calcareous soils having the worst performance on volcanic soils and seeds from hybrids performing badly on calcareous soils and most probably worse than the other seed classes (Fig. 3c). These results contrast to those of the UK experiment, for which there was no difference in germination between the four seed sources, and survival of hybrid seedlings was similar to that of *H. forsteriana* (Fig. S2).

**Table 4:** The distribution of *P*‐values from models to assess post‐zygotic isolation by comparing the effects of seed source and soil type on germination and survival of hybrids to pure species, using an under‐sampling method to account for unbalanced sample sizes

Effect	Min *P*	Q1 *P*	Median *P*	Mean *P*	Q3 *P*	Max *P*
Soil type	0.000	0.330	0.598	0.552	0.794	1.000
Seed source	0.000	0.085	0.318	0.375	0.618	0.998
Soil type × Seed source	0.000	0.015	0.059	0.184	0.239	0.999

Twenty‐four replicate data points for the three pure species classes were sampled 1000 times and the variation in germination and survival tested against the hybrid class. The minimum, lower quartile, median, mean, upper quartile and maximum *P*‐values for the effect of seed origin, soil and their interaction are reported.

## Discussion

The hypothesized ecological speciation scenario of *Howea* palms on LHI was based on currently observed displaced flowering times and displaced edaphic distributions between the two species (Savolainen *et al*., 2006). However, how divergent selection associated with soil type adaptation and differences in flowering phenologies interacted to drive speciation remained unclear. This study provides elements of the intensity of pre‐zygotic isolation, its consistency with the rare occurrence of hybrids on the island and indications of both adaptation to soil type and post‐zygotic isolation at seedlings stage.

### Establishment of a genetic component to flowering

Previous estimates of flowering time and displacement between the two *Howea* species were obtained in many sites across LHI (Savolainen *et al*., 2006) and could therefore not address the role of plastic vs. genetic components to observations. Monitoring the phenology of about 50 trees of each species growing *in situ* and in *ex situ* common gardens, we show that the displacement of flowering phenologies between *H. belmoreana* and *H. forsteriana* is stable across environmental gradients, with *H. forsteriana* consistently flowering earlier in the season than *H. belmoreana*. This result, along with the observation that genetically identified hybrid trees have an intermediate flowering phenology between the two parental species, establishes that flowering in *Howea* is, in part, genetically determined. These data also show that flowering is partially controlled by the environment, as shown in other studies (Mazer & Schick, 1991; Antonovics, 2006; Franks *et al*., 2007): we observed differences in the onset and duration of flowering and differences in protandry for each species between the two common gardens (Fig. 2). We were, however, unable to find which environmental variables, among the climatic and edaphic ones, are responsible for this plasticity.

### Strong pre‐zygotic reproductive isolation

Overall current levels of pre‐zygotic isolation measured on LHI are of the same magnitude as those measured for other plant species (Rieseberg & Willis, 2007; Widmer *et al*., 2009). Pre‐zygotic isolation between the two species via the sole displacement of flowering phenologies reduced potential gene flow from *H. forsteriana* to *H. belmoreana* by 97% (compared with full synchrony) and from *H. belmoreana* to *H. forsteriana* by 80%. The same estimates using the phenological data of Savolainen *et al*. (2006) measured across multiple sites on LHI are similar, with a reproductive isolation at the species level of 0.98 for *H. belmoreana* and 0.81 for *H. forsteriana*, indicating the stability of both the intensity and direction of gene flow. This stability is explained by *H. forsteriana* consistently flowering earlier and consistently being protandrous on LHI. Although pre‐zygotic barriers are often reported to contribute more than post‐zygotic barriers to reproductive isolation in plants (Rieseberg & Willis, 2007; Lowry *et al*., 2008), these studies have most often been conducted on short‐lived species. The contribution of pre‐ vs. post‐zygotic isolation mechanisms could be different for long‐lived species, but too few data are currently available to conclude. Examples in a group of four species of *Cyrtandra* show pre‐zygositc isolation through flowering phenology was the largest component of reproductive isolation for one species, whereas post‐zygotic barriers were more important for the remaining three species (Johnson *et al*., 2015).

We find that the dichogamy (i.e. protandry) estimate developed here and applied to the previous data provide similar results: protandry at the species level is higher (0.18 here compared to 0.22 in 2006) for *H. forsteriana* than for *H. belmoreana* (0.04 for both years). Plasticity in the displacement of male vs. female flowering in *H. forsteriana* appears adaptive on LHI, reinforcing the barrier to gene flow between the two species. However, using the data collected across LHI (Savolainen *et al*., 2006), protandry at the population level of *H. forsteriana* did not replicate over years in the same location: *H. forsteriana* trees monitored in the Far Flats (same location as for the data reported in this study) were mostly protogynous (with female flowers open before male flowers) in Savolainen *et al*. (2006) but protandrous (with male flowers open before female flowers) in 2009. Dichogamy of *H. forsteriana* also did not correlate with soil, but variation of dichogamy was lower on calcareous soils (SD = 0.06 for five locations) than it was on volcanic soils (SD = 0.28 for four locations).

### Evidence of post‐zygotic reproductive isolation

Despite strong pre‐zygotic reproductive isolation, some adult hybrids are found on LHI, with a total of 19 morphologically intermediate trees confirmed genetically as hybrids over dozens of field trips (Savolainen *et al*., 2006; Babik *et al*., 2009; Papadopulos *et al*., 2013). These numbers are evidently underestimates of hybridization at the adult stage because (i) some parts of the island where the two species grow are inaccessible, (ii) tree density can be high in some locations (about three trees in only 1 m²), thus decreasing the detection power, and (iii) we are better at detecting F1 hybrids, from leaf intermediate morphology, than detecting hybrids of later generations (Table S1). Because of these limitations, combined with our strategy to search for hybrids only in locations where the two species grow in close vicinity, we cannot provide an accurate estimate of the frequency of hybrids over LHI. Yet, from a rough estimate of ~ 100 000 *Howea* trees of the two species on the LHI (C. Devaux & V. Savolainen unpublished data) and the efforts we made to look for hybrids, we can confidently state that adult hybrids are rare and occur at a very low frequency, consistently with our estimate of pre‐zygotic isolation.

We further find here that hybrids at the seedling stage occur at a higher frequency than do hybrids at the adult stage: about 7% of the seedlings, sampled in two locations on LHI in which we expect hybridization, were hybrids of first or later generations. A similar pattern has been observed in oaks, for which 80% more hybrids were found in the seedling stage rather than in adults, indicating that selection against hybrids takes place prior to maturity (Curtu *et al*., 2009). This is a greater difference than the one measured in *Howea*, although our estimate of hybridization at the seeding stage may be underestimated due to technical difficulties in detecting hybrids of generations later than four with the resolution of our AFLP data. For example, we could not assign 13% of the seedling collected in the Far Flats where the two species grow intermingled and where several adult hybrids of late generations were found. We expected in this population to find progeny of higher generations, which we unfortunately did not detect with our method. In the site in which the two species grow adjacently, we assigned more individuals to hybrid classes and they were of at least of the second generation. Finding these hybrid seedlings informs us that we missed some hybrids at the adult stage.

The lower occurrence of hybrids at a late, that is adult, vs. early, that is seedling, stage indicates that selection must act between these two stages, but that post‐zygotic isolation is not complete. More empirical data are needed to uncover the exact stages and traits that are the source of post‐zygotic isolation. A proposed common mechanism for our observed pattern is the reduction in fitness of F2 hybrids compared with the parental species due to mismatches in several functional traits, underpinned by multiple genomic regions [e.g. in sticklebacks, Arnegard *et al*., 2014). Examining gene expression in *Howea* F1 hybrids (L. Dunning & V. Savolainen, unpublished data)] found some evidence for gene misexpression, which can lead to maladapted phenotypes and reduced fitness of hybrids (Renaut *et al*., 2009). Gene misexpression and hybrid breakdown were also observed in F2 hybrids in *Senecio*, involving reduced expression for genes controlling growth and developmental pathways (Chapman *et al*., 2016).

We found some lines of evidence of post‐zygotic isolation with the results of the seed transplant experiment on LHI, which also allowed estimation of early fitness components. However, we cannot report here significant results, but only trends, as we calculated that at least three times more seeds should have been planted to detect significant differences in survival of hybrid vs. pure parental species. Seeds collected on genetically confirmed F1 hybrid trees tended to germinate and survive as well as *H. belmoreana* trees (which are specialist of volcanic soils) on volcanic soils. In contrast, the number of seeds from F1 hybrids alive at month 36 was smaller than that of *H. belmoreana* or *H. forsteriana* when planted on calcareous soils. These trends match our observation of the absence of hybrid adults on calcareous soils (Fig. 1, we are not able to morphologically discriminate pure species from hybrids at seedling stage) and suggest again that post‐zygotic selection occurs at a later stage than the one measured in this study. Few long‐term studies on hybrid survival and reproduction are now published to evaluate the generality or singularity of our results with those of others. Hybrid seedlings in oaks were monitored for 4 years and had a lower survival than that of their parental species (Lepais *et al*., 2013). A study on *Eucalyptus* showed abnormal phenotypes for hybrids only after 2 years and that these individuals usually died before reaching maturity (Potts & Dungey, 2004).

### Local adaptation at an early stage

The reciprocal seed transplant also allowed us to evaluate local adaptation of the two *Howea* species on the island; a power analysis confirms that the sample sizes used for this study were enough to detect differences between the three types of seeds we planted. Our measures combined both seed viability and germination, which can therefore not be distinguished. Nevertheless, seeds of *H. forsteriana*, which is much studied because of its commercial value, have been shown to have 100% viability when freshly harvested (Chin *et al*., 1988); we have no information on *H. belmoreana* or hybrids seed viability. We observed delayed germination both in the field and in the UK but cannot evaluate whether this is due to seed dormancy, neither whether seed dormancy can explain the low rate of germination in the LHI experiment. The delay in germination is commonly observed for *H. forsteriana* in the palm seed nursery on LHI: seeds start to germinate three months after planting under controlled conditions. We also know from the nursery that *H. forsteriana* seeds can still germinate two years after planting, meaning they can survive that long, and that overall germination is at a high rate (70%) under controlled conditions (Larry Wilson, pers. comm.). We have no information on seed germination or dormancy for *H. belmoreana* or for either species under field conditions, except from this study. We suspect from our observations scattered through many field trips that seed germination may be low under natural conditions and that the establishment of a seed bank is unlikely (we found very few seeds underground when we dug up soil for setting up 140 cages in 14 different sites).

We controlled in this experiment the soil type on which the seeds were collected: on volcanic soils for *H. belmoreana* and *H. forsteriana* and on calcareous for *H. forsteriana* only, as no *H. belmoreana* tree currently grows on this soil type on the island. Given the absence of *H. belmoreana* on calcareous soils as mature trees, it was surprising that seeds collected on *H. belmoreana* performed as well on calcareous as on volcanic soils: estimations of germination, survival and height of seedlings were unaffected by soil type (Fig. 3). We conclude that selection may be acting against *H. belmoreana* at a later stage, between the juvenile stage and maturity, as hypothesized for hybrid palms. However, given the long life span of *Howea* (~ 10 years to reach maturity) it would be a great experimental challenge to determine when and how selection takes place. Trees are also exposed to a high diversity of biotic and abiotic conditions (e.g. likelihood to experience extreme weather) compared with short‐lived species, and evaluating selection acting on them is thus complex because it may be partitioned in many components that are difficult to tease apart (Petit & Hampe, 2006). Seedlings from *H. forsteriana* trees growing on calcareous and volcanic soils had similar early fitness components. The ranking of seeds types for early fitness components on volcanic soils was as expected: *H. belmoreana* seedlings performed much better than did *H. forsteriana* seedlings*, and H. forsteriana* seedlings from trees growing on volcanic soils performed much better than did *H. forsteriana* seedlings from trees growing on calcareous soils. Overall, these results point to local adaptation at an early stage.

Differences occurred in the results of the transplant experiments between LHI and the UK. First, although we did our best to mimic the native soils, those artificial soils used in the UK must have different chemical composition to that of LHI. The compost used in the glasshouses is probably more nutrient rich, which would explain the high germination rate of both species. Secondly, the conditions in glasshouses are more favourable to germination, for example without drought, in contrast to what happened on LHI during the experiment.

Despite evidence for local adaptation at an early stage, no clear genetic structure across soil types within *H. forsteriana* has been detected: Dunning *et al*. (2016) found no evidence for genetic structure based on examining 22 741 transcriptome‐derived SNPs, whereas Babik *et al*. (2009) found limited evidence for two genetic clusters based on 625 AFLP markers, although this differentiated between the most southerly populations and more northern ones, rather than between the two soil types. Early local adaptation to calcareous soils in *H. forsteriana* may be driven by transgenerational epigenetic effects rather than background genetic differentiation. Functionally adaptive transgenerational effects due to environmental stresses have been shown to advantageously alter germination and survival rates in *Bromus tectorum* (Meyer & Allen, 1999) and *Arabidopsis thaliana* (Boyko *et al*., 2010). Although there is no evidence for genetic structure within *H. forsteriana*, there is clear genetic differentiation between the species, with 1320 genes of 14 576 examined showing fixed polymorphisms (Dunning *et al*., 2016). Many of these genes are involved in processes such as water deprivation and phosphate starvation, suggesting adaptation to calcareous soils and subsequent recolonization of volcanic soils after fixation (Dunning *et al*., 2016).

## Conclusion

Reproductive isolation and divergent selection are two fundamental elements of ecological speciation. In *Howea*, we show that the displacement of flowering phenologies between the two species is partly under genetic control and dramatically reduces gene flow between species. Two lines of evidence show that post‐zygotic isolation occurs but is not complete: hybridization at the seedling stage is lower than at the adult stage, and hybrid seedlings planted in a controlled experimental design tended to survive less than the parental species on calcareous soils.

## Supporting information


**Figure S1** Temporal dynamic of germination (cumulative germination shown for the duration of the experiments) according to seed source (triangles for *Howea belmoreana*, circles for *H. forsteriana* from volcanic soil, squares for *H. forsteriana* from calcareous soil) and seeding soil (full symbols for volcanic and open symbols for calcareous) for the seed transplant experiment on LHI (panel a) and in the UK (panel b).

**Figure S2** Germination and survival of seeds and seedlings across mimicked soil types from the reciprocal transplant experiment in the UK.

**Table S1** Excel table of AFLP genotyping results and hybrid classifications.

**Table S2** Experimental design for finding determinants of seed germination under controlled conditions in the UK glasshouse experiment.
**Table S3** Results of the generalized mixed models to test the effects of seed origin, UK soil, glasshouse planting position, seed density and the interactions between seed origin and UK soil and seed density on germination and seedling survival in the UK. Wald F test statistic values are reported, along with the degrees of freedom (DF) and associated *P* values.
**Table S4** LogLikelihood of several models testing for the effect of seed source (origin), soil and their interaction, as well as and for species, soil and their interaction on the temporal dynamic of germination for the LHI experiment.
**Table S5** LogLikelihood of several models testing for the effect of seed source (origin), soil and their interaction, as well as and for species, soil and their interaction on the temporal dynamic of germination for the UK experiment.

## References

[jeb12933-bib-0001] Anderson, E.C. & Thompson, E.A. 2002 A model‐based method for identifying species hybrids using multilocus genetic data. Genetics 160: 1217–1229. 11901135 10.1093/genetics/160.3.1217 PMC1462008

[jeb12933-bib-0002] Antonovics, J. 2006 Evolution in closely adjacent plant populations X: long‐term persistence of prereproductive isolation at a mine boundary. Heredity 97: 33–37. 16639420 10.1038/sj.hdy.6800835

[jeb12933-bib-0003] Arnegard, M.E. , McGee, M.D. , Matthews, B. , Marchinko, K.B. , Conte, G.L. , Kabir, S. *et al* 2014 Genetics of ecological divergence during speciation. Nature 511: 307–311. 24909991 10.1038/nature13301 PMC4149549

[jeb12933-bib-0004] Babik, W. , Butlin, R.K. , Baker, W.J. , Papadopulos, A.S.T. , Boulesteix, M. , Anstett, M.‐C. *et al* 2009 How sympatric is speciation in the *Howea* palms of Lord Howe Island? Mol. Ecol. 18: 3629–3638. 19674301 10.1111/j.1365-294X.2009.04306.x

[jeb12933-bib-0005] Barluenga, M. , Stölting, K.N. , Salzburger, W. , Muschick, M. & Meyer, A. 2006 Sympatric speciation in Nicaraguan crater lake cichlid fish. Nature 439: 719–723. 16467837 10.1038/nature04325

[jeb12933-bib-0006] Boyko, A. , Blevins, T. , Yao, Y. , Golubov, A. , Bilichak, A. , Ilnytskyy, Y. *et al* 2010 Transgenerational adaptation of *Arabidopsis* to stress requires DNA methylation and the function of dicer‐like proteins. PLoS One 5: e9514. 20209086 10.1371/journal.pone.0009514 PMC2831073

[jeb12933-bib-0007] Chapman, M.A. , Hiscock, S.J. & Filatov, D.A. 2016 The genomic bases of morphological divergence and reproductive isolation driven by ecological speciation in *Senecio* (Asteraceae). J. Evol. Biol. 29: 98–113. 26414668 10.1111/jeb.12765

[jeb12933-bib-0008] Chin, H.F. , Krishnapillay, B. & Alang, Z.C. 1988 Breaking dormancy in kentia palm seeds by infusion technique. Pertanika 11: 137–141.

[jeb12933-bib-0009] Coyne, J.A. & Orr, H.A. 2004 Speciation. Sinauer Associates, Massachusetts.

[jeb12933-bib-0010] Curtu, A.L. , Gailing, O. & Finkeldey, R. 2009 Patterns of contemporary hybridization inferred from paternity analysis in a four‐oak‐species forest. BMC Evol. Biol. 9: 284. 19968862 10.1186/1471-2148-9-284 PMC2795763

[jeb12933-bib-0011] Devaux, C. & Lande, R. 2008 Incipient allochronic speciation due to non‐selective assortative mating by flowering time, mutation and genetic drift. Proc. R. Soc. B Biol. Sci. 275: 2723–2732. 10.1098/rspb.2008.0882 PMC2605824 18700202

[jeb12933-bib-0012] Devaux, C. & Lande, R. 2009 Displacement of flowering phenologies among plant species by competition for generalist pollinators. J. Evol. Biol. 22: 1460–1470. 19467129 10.1111/j.1420-9101.2009.01762.x

[jeb12933-bib-0013] Dieckmann, U. & Doebeli, M. 1999 On the origin of species by sympatric speciation. Nature 400: 354–357. 10432112 10.1038/22521

[jeb12933-bib-0014] Dunning, L.T. , Hipperson, H. , Baker, W.J. , Butlin, R.K. , Devaux, C. , Hutton, I. *et al* 2016 Ecological speciation in sympatric palms: 1. Gene expression, selection and pleiotropy. J. Evol. Biol. 29: 1472–1487. 27177130 10.1111/jeb.12895 PMC6680112

[jeb12933-bib-0015] Franks, S.J. , Sim, S. & Weis, A.E. 2007 Rapid evolution of flowering time by an annual plant in response to a climate fluctuation. Proc. Natl. Acad. Sci. U.S.A. 104: 1278–1282. 17220273 10.1073/pnas.0608379104 PMC1783115

[jeb12933-bib-0016] Gavrilets, S. & Vose, A. 2007 Case studies and mathematical models of ecological speciation. 2. Palms on an oceanic island. Mol. Ecol. 16: 2910–2921. 17614906 10.1111/j.1365-294X.2007.03304.x

[jeb12933-bib-0017] Hoekstra, H.E. & Coyne, J.A. 2007 The locus of evolution: evo devo and the genetics of adaptation. Evolution 61: 995–1016. 17492956 10.1111/j.1558-5646.2007.00105.x

[jeb12933-bib-0018] Johnson, M.A. , Price, D.K. , Price, J.P. & Stacy, E.A. 2015 Postzygotic barriers isolate sympatric species of *Cyrtandra* (Gesneriaceae) in Hawaiian montane forest understories. Am. J. Bot. 102: 1870–1882. 26542848 10.3732/ajb.1500288

[jeb12933-bib-0019] Lepais, O. , Roussel, G. , Hubert, F. , Kremer, A. & Gerber, S. 2013 Strength and variability of postmating reproductive isolating barriers between four European white oak species. Tree Genet. Genomes 9: 841–853.

[jeb12933-bib-0020] Lowry, D.B. , Modliszewski, J.L. , Wright, K.M. , Wu, C.A. & Willis, J.H. 2008 The strength and genetic basis of reproductive isolating barriers in flowering plants. Philos. Trans. R. Soc. Lond. B Biol. Sci. 363: 3009–3021. 18579478 10.1098/rstb.2008.0064 PMC2607309

[jeb12933-bib-0021] Martin, N.H. & Willis, J.H. 2007 Ecological divergence associated with mating system causes nearly complete reproductive isolation between sympatric *Mimulus* species. Evolution 61: 68–82. 17300428 10.1111/j.1558-5646.2007.00006.x

[jeb12933-bib-0022] Martin, S.H. , Dasmahapatra, K.K. , Nadeau, N.J. , Salazar, C. , Walters, J.R. , Simpson, F. *et al* 2013 Genome‐wide evidence for speciation with gene flow in *Heliconius* butterflies. Genome Res. 23: 1817–1828. 24045163 10.1101/gr.159426.113 PMC3814882

[jeb12933-bib-0023] Mazer, S.J. & Schick, C.T. 1991 Constancy of population parameters for life history and floral traits in *Raphanus sativus* L. I. Norms of reaction and the nature of genotype by environment interactions. Heredity 67: 143–156. 10.1111/j.1558-5646.1991.tb02694.x 28563970

[jeb12933-bib-0024] Meyer, S.E. & Allen, P.S. 1999 Ecological genetics of seed germination regulation in *Bromus tectorum* L. II. Reaction norms in response to a water stress gradient imposed during seed maturation. Oecologia 120: 35–43. 10.1007/s004420050830 28308051

[jeb12933-bib-0025] Milne, R.I. & Abbott, R.J. 2008 Reproductive isolation among two interfertile *Rhododendron* species: low frequency of post‐F1 hybrid genotypes in alpine hybrid zones. Mol. Ecol. 17: 1108–1121. 18261051 10.1111/j.1365-294X.2007.03643.x

[jeb12933-bib-0026] Nosil, P. 2012 Ecological Speciation. Oxford University Press, Oxford.

[jeb12933-bib-0027] Papadopulos, A.S.T. , Price, Z. , Devaux, C. , Hipperson, H. , Smadja, C.M. , Hutton, I. *et al* 2013 A comparative analysis of the mechanisms underlying speciation on Lord Howe Island. J. Evol. Biol. 26: 733–745. 23320532 10.1111/jeb.12071

[jeb12933-bib-0028] Papadopulos, A.S.T. , Kaye, M. , Devaux, C. , Hipperson, H. , Lighten, J. , Dunning, L.T. *et al* 2014 Evaluation of genetic isolation within an island flora reveals unusually widespread local adaptation and supports sympatric speciation. Philos. Trans. R. Soc. Lond. B Biol. Sci. 369: 20130342. 24958917 10.1098/rstb.2013.0342 PMC4071517

[jeb12933-bib-0029] Petit, R.J. & Hampe, A. 2006 Some evolutionary consequences of being a tree. Annu. Rev. Ecol. Evol. Syst. 37: 187–214.

[jeb12933-bib-0030] Potts, B.M. & Dungey, H.S. 2004 Interspecific hybridisation of *Eucalyptus*: key issues for breeders and geneticists. New Forest. 27: 115–138.

[jeb12933-bib-0031] Renaut, S. , Nolte, A.W. & Bernatchez, L. 2009 Gene expression divergence and hybrid misexpression between lake whitefish species pairs (*Coregonus* spp. Salmonidae). Mol. Biol. Evol. 26: 925–936. 19174479 10.1093/molbev/msp017

[jeb12933-bib-0032] Rieseberg, L.H. & Willis, J.H. 2007 Plant speciation. Science 317: 910–914. 17702935 10.1126/science.1137729 PMC2442920

[jeb12933-bib-0033] Rundle, H.D. & Nosil, P. 2005 Ecological speciation. Ecol. Lett. 8: 336–352.

[jeb12933-bib-0034] Savolainen, V. , Anstett, M.‐C. , Lexer, C. , Hutton, I. , Clarkson, J.J. , Norup, M.V. *et al* 2006 Sympatric speciation in palms on an oceanic island. Nature 441: 210–213. 16467788 10.1038/nature04566

[jeb12933-bib-0035] Schluter, D. 2000 The Ecology of Adaptive Radiation. Oxford University Press, Oxford.

[jeb12933-bib-0036] Schluter, D. 2001 Ecology and the origin of species. Trends Ecol. Evol. 16: 372–380. 11403870 10.1016/s0169-5347(01)02198-x

[jeb12933-bib-0037] Schluter, D. 2009 Evidence for ecological speciation and its alternative. Science 323: 737–741. 19197053 10.1126/science.1160006

[jeb12933-bib-0038] Smadja, C.M. & Butlin, R.K. 2011 A framework for comparing processes of speciation in the presence of gene flow. Mol. Ecol. 20: 5123–5140. 22066935 10.1111/j.1365-294X.2011.05350.x

[jeb12933-bib-0039] Stam, P. 1983 The evolution of reproductive isolation in closely adjacent plant populations through differential flowering time. Heredity 50: 105–118.

[jeb12933-bib-0040] Widmer, A. , Lexer, C. & Cozzolino, S. 2009 Evolution of reproductive isolation in plants. Heredity 102: 31–38. 18648386 10.1038/hdy.2008.69

