# Supplementary figures and images for: Ecological speciation in sympatric palms: 2. Pre‐ and post‐zygotic isolation
Source: J Evol Biol. 2016 Jul 20;29(11):2143–56. doi: 10.1111/jeb.12933 (PMC5096058; doi:10.1111/jeb.12933)

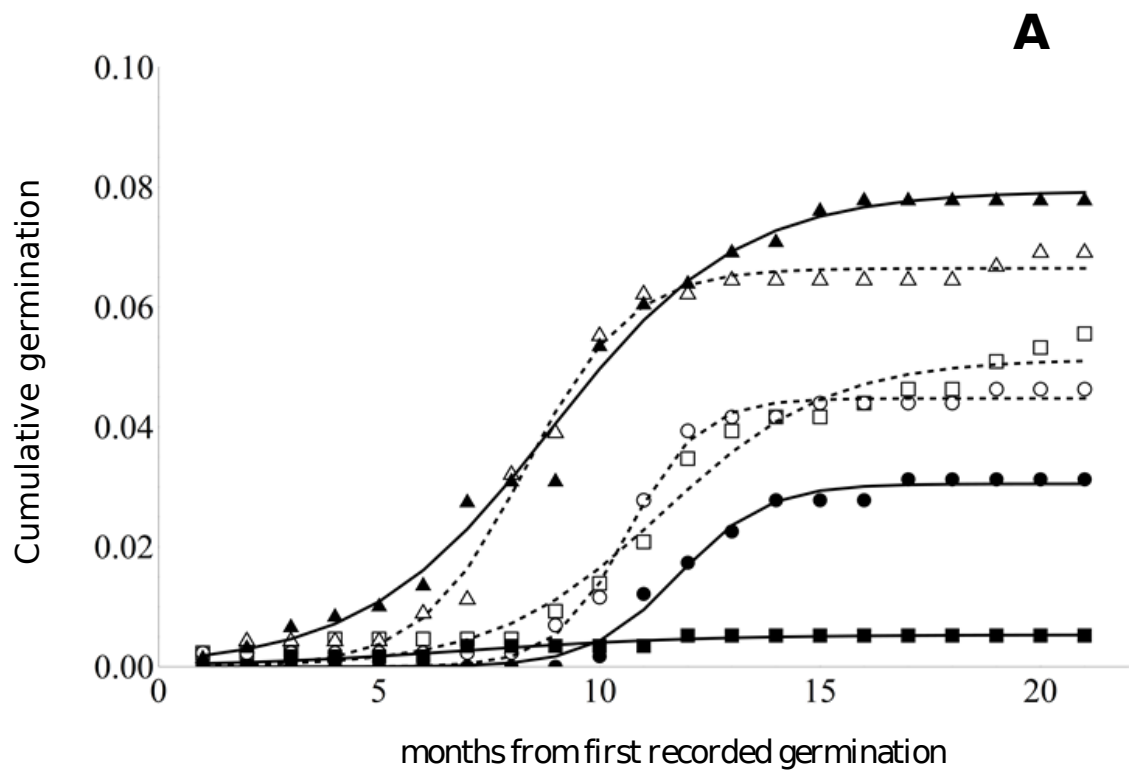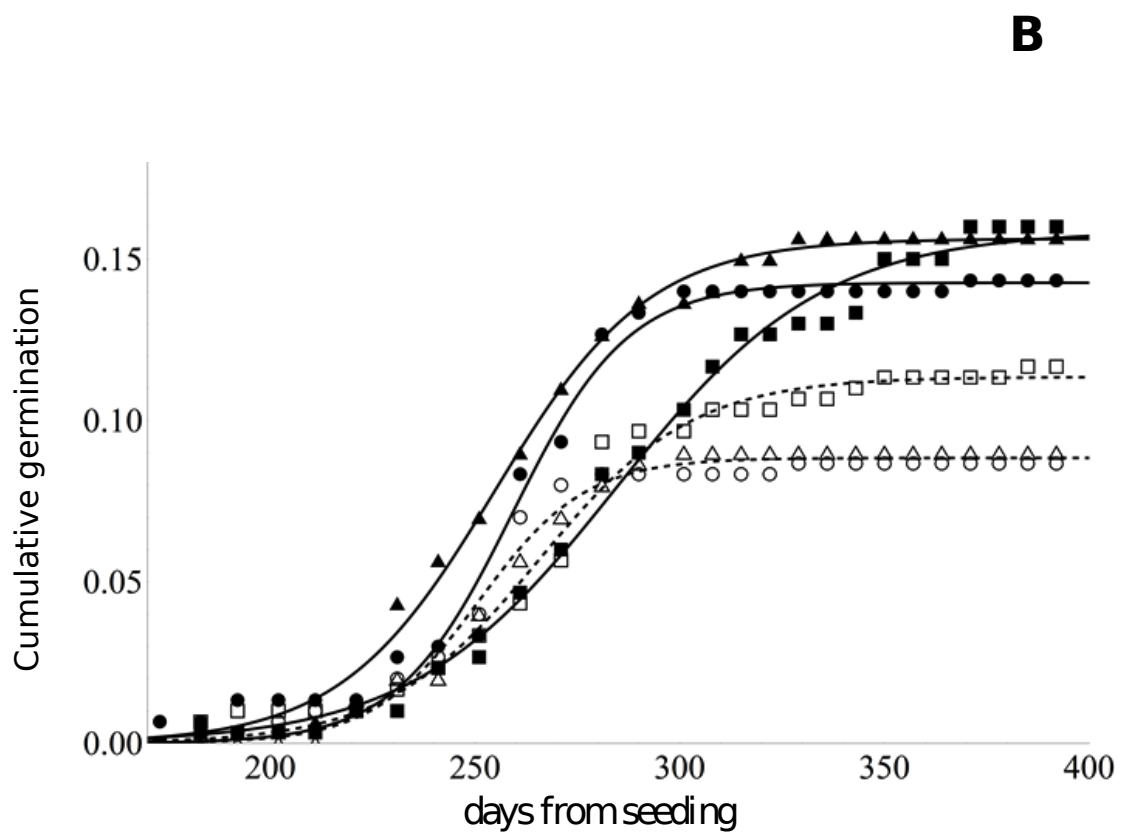

Supplement: Supplementary file 1 — Figure S1 Temporal dynamic of germination (cumulative germination shown for the duration of the experiments) according to seed source (triangles for Howea belmoreana, circles for H. forsteriana from volcanic soil, squares for H. forsteriana from calcareous soil) and seeding soil (full symbols for volcanic and open symbols for calcareous) for the seed transplant experiment on LHI (panel a) and in the UK (panel b). [file JEB-29-2143-s001.pdf]

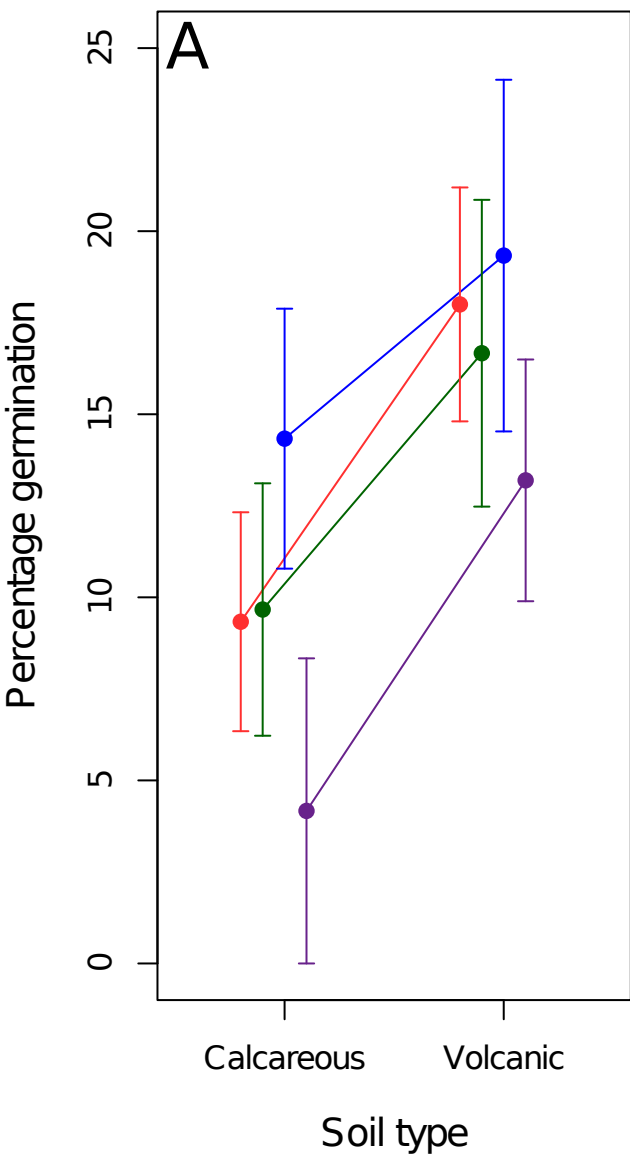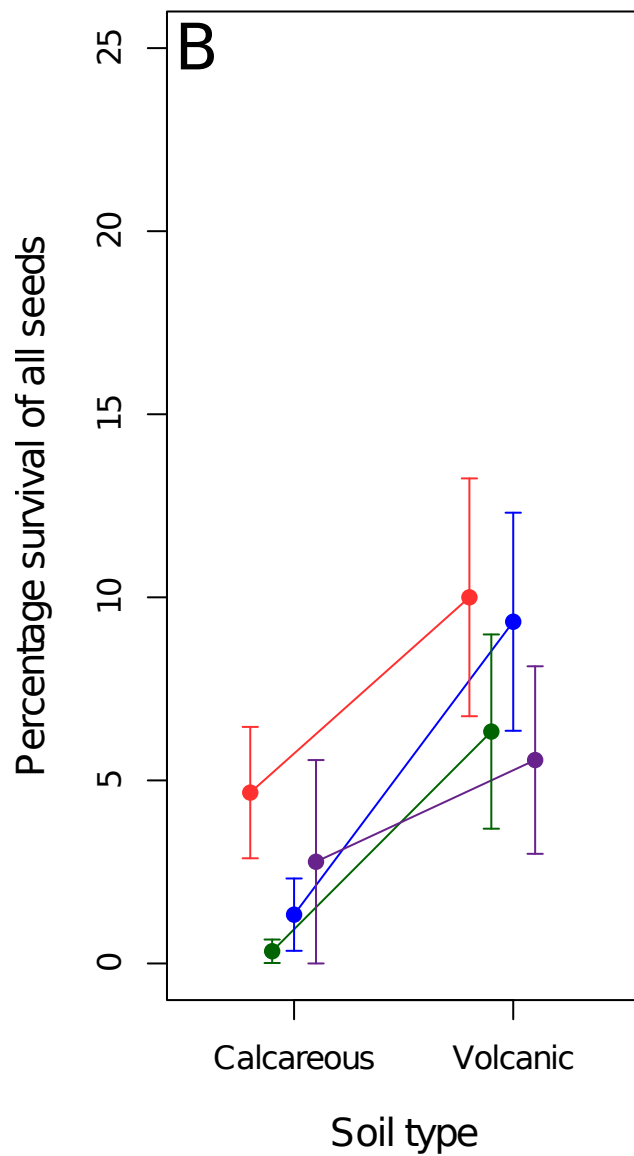

Supplement: Supplementary file 2 — Figure S2 Germination and survival of seeds and seedlings across mimicked soil types from the reciprocal transplant experiment in the UK. [file JEB-29-2143-s002.pdf]
