# Supplementary material for: Ecological speciation in sympatric palms: 2. Pre‐ and post‐zygotic isolation
Source: J Evol Biol. 2016 Jul 20;29(11):2143–56. doi: 10.1111/jeb.12933 (PMC5096058; doi:10.1111/jeb.12933)
Supplement: Supplementary file 4 — Table S2 Experimental design for finding determinants of seed germination under controlled conditions in the UK glasshouse experiment. Table S3 Results of the generalized mixed models to test the effects of seed origin, UK soil, glasshouse planting position, seed density and the interactions between seed origin and UK soil and seed density on germination and seedling survival in the UK. Wald F test statistic values are reported, along with the degrees of freedom (DF) and associated P values. Table S4 LogLikelihood of several models testing for the effect of seed source (origin), soil and their interaction, as well as and for species, soil and their interaction on the temporal dynamic of germination for the LHI experiment. Table S5 LogLikelihood of several models testing for the effect of seed source (origin), soil and their interaction, as well as and for species, soil and their interaction on the temporal dynamic of germination for the UK experiment. [file JEB-29-2143-s004.docx]

**SUPPLEMENTARY INFORMATION**

**Ecological speciation in sympatric palms: 2. Pre- and post-zygotic isolation**

H. HIPPERSON^*,1,2^, L. T. DUNNING^*,1,2^, W. J. BAKER^†^, R. K. BUTLIN^§^, I. HUTTON^¶^, A. S. T. PAPADOPULOS^*,†^, C. M. SMADJA^*,3^, T. C. WILSON^¥^, C. DEVAUX^3,4^, V. SAVOLAINEN^*,†,4,5^

**Legend of Supplementary Figures**

**Figure S1**. Temporal dynamic of germination (cumulative germination shown for the duration of the experiments) according to seed source (triangles for *H. belmoreana*, circles for *H. forsteriana* from volcanic soil, squares for *H. forsteriana* from calcareous soil) and seeding soil (full symbols for volcanic and open symbols for calcareous) for the seed transplant experiment on LHI (panel A) and in the UK (panel B).

**Figure S2**. Germination and survival of seeds and seedlings across mimicked soil types from the reciprocal transplant experiment in the UK. Percentage germination (panel A), survival of all seeds (panel B), *H. belmoreana* (*B*, red), *H. forsteriana* from calcareous soils (*FC*, blue), *H. forsteriana* from volcanic soils (*FV*, green), hybrids (*H*, purple). Each point represents the mean (±SE) percentage of seeds germinated (panel A) or survived (panel B) across replicate planting trays of mimicked calcareous (C) and volcanic (V) soil. Sample sizes for germinated seeds (panel A): *B* = 82 (28 C, 54 V), *FC* = 101 (43 C, 58 V), *FV* = 79 (29 C, 50 V), *H* = 25 (6 C, 19 V); and for survived seeds (panel B): *B* = 44 (14 C, 30 V), *FC* = 32 (4 C, 28 V), *FV* = 20 (1 C, 19 V), *H* = 12 (4 C, 8 V).

**Supplementary Tables**

**Table S1**. Excel table of AFLP genotyping results and hybrid classifications.

**Table S2**. Experimental design for finding determinants of seed germination under controlled conditions in the UK glasshouse experiment.

| Seed sources |  | Hybrids from  volcanic soil | | | *H. belmoreana*  from volcanic soil | | | *H. forsteriana*  from calcareous soil | | | *H. forsteriana*  from volcanic soil | | |
| --- | --- | --- | --- | --- | --- | --- | --- | --- | --- | --- | --- | --- | --- |
| Blocks of 30 trays |  | A | B | C | A | B | C | A | B | C | A | B | C |
| Seeding soil and number of seeds | ‘volcanic’ | 24 | 24 | 96 | 84 | 120 | 96 | 108 | 72 | 120 | 132 | 48 | 120 |
|  | ‘calcareous’ | 72 | 0 | 72 | 84 | 96 | 120 | 72 | 132 | 96 | 168 | 60 | 72 |
| Total number of seeds |  | 96 | 24 | 168 | 168 | 216 | 216 | 180 | 204 | 216 | 300 | 108 | 192 |

**Table S3.** Results of the generalized mixed models to test the effects of seed origin, UK soil, glasshouse planting position, seed density and the interactions between seed origin and UK soil and seed density on germination and seedling survival in the UK. Wald F test statistic values are reported, along with the degrees of freedom (DF) and associated *P* values. *P* < 0.05 are highlighted in bold. Replicate planting trays were modelled as a random effect, and their size is also reported.

|  | **Germination**^1^ | | | **Survival**^2^ | | |
| --- | --- | --- | --- | --- | --- | --- |
| **Effect** | **F** | **DF** | ***P*** | **F** | **DF** | ***P*** |
| Seed source | 0.98 | 2 | 0.376 | 3.66 | 2 | **0.028** |
| UK soil type | 4.35 | 1 | **0.037** | 8.78 | 1 | **0.004** |
| Seed source x UK soil type | 0.36 | 2 | 0.695 | 1.16 | 2 | 0.317 |
| Spatial block | 4.72 | 2 | **0.009** | 3.29 | 2 | **0.040** |
| Position within blocks | 7.32 | 1 | **0.007** | 1.36 | 1 | 0.246 |
| Seed density | 7.86 | 1 | **0.005** | 0.01 | 1 | 0.918 |
| Seed density x Seed source | 14.11 | 2 | **<0.001** | 0.12 | 2 | 0.890 |

^1^ Random effect estimate of replicate trays (and standard error) = 0.971 (0.278)

^2^ Random effect estimate of replicate trays (and standard error) = 1.517 (0.717)

Table S4. LogLikelihood of several models testing for the effect of seed source (origin), soil and their interaction, as well as and for species, soil and their interaction on the temporal dynamic of germination for the LHI experiment. All p-values of likelihood ratio tests were close to 0, except when testing if FC grew similarly on calcareous and volcanic soils (the p-value for accepting the nested model is given in bold).

| Origin | Soil | model :  origin × soil | model :  origin | Origin | Soil | model :  soil | model :  species×soil | Origin | Soil | model :  type × soil | model :  soil × soil |
| --- | --- | --- | --- | --- | --- | --- | --- | --- | --- | --- | --- |
| *FV* | C | -24.55 | -33.82 | FV | V | -45.52 | -25.74 | FV | V | -22.76 | -44.07 |
| *FV* | V | -22.76 |  | FC |  |  |  | B | V | -40.35 |  |
| *FC* | C | -2.18 | **-36.87 (p=0.978)** | BV |  |  | -40.35 | FC | V | -34.6 | -34.6 |
| *FC* | V | -34.6 |  | FV | C | -48.90 | -45.35 | FV | C | -24.55 | -38.9 |
| *B* | C | -32.39 | -46.47 | FC |  |  |  | B | C | -32.39 |  |
| *B* | V | -40.35 |  | BV |  |  | -32.39 | FC | C | -2.18 | -2.18 |

Table S5. LogLikelihood of several models testing for the effect of seed source (origin), soil and their interaction, as well as and for species, soil and their interaction on the temporal dynamic of germination for the UK experiment. All p-values of likelihood ratio tests were close to 0, except when testing if B and FV grew similarly on calcareous soil (the p-value for accepting the nested model is given in bold).

| Origin | Soil | model :  origin × soil | model :  origin | Origin | Soil | model :  soil | model :  species×soil | Origin | Soil | model :  type × soil | model :  soil × soil |
| --- | --- | --- | --- | --- | --- | --- | --- | --- | --- | --- | --- |
| *FV* | C | 50.82 | 24.91 | FV | V | 92.23 | 66.53 | FV | V | 44.59 | 83.14 |
| *FV* | V | 44.59 |  | FC |  |  |  | B | V | 59.79 |  |
| *FC* | C | 46.98 | 59.265 | B |  |  | 59.79 | FC | V | 55.1 | 55.1 |
| *FC* | V | 55.1 |  | FV | C | 94.62 | 55.39 | FV | C | 50.82 | **108.31 (p=0.067)** |
| *B* | C | 61.07 | 21.65 | FC |  |  |  | B | C | 61.07 |  |
| *B* | V | 59.79 |  | B |  |  | 61.7 | FC | C | 46.98 | 46.98 |
